# Optimal feature encoding in early vision

**DOI:** 10.1038/s41598-025-07644-9

**Published:** 2025-07-03

**Authors:** Serena Castellotti, Giacomo Mazzotta, Alessandro Benedetto, Maria Michela Del Viva

**Affiliations:** 1 Department of Physics, University of Pisa, Pisa, Italy; 2 Department of Translational Research on New Technologies in Medicine and Surgery, University of Pisa, Pisa, Italy; 3 Department of Neurosciences, Psychology, Drug Research and Child Health (NEUROFARBA), University of Florence, Florence, Italy

**Keywords:** Fast vision, Efficient coding, Information maximization, EEG, VEP, C1 component, Cognitive neuroscience, Computational neuroscience, Sensory processing, Visual system

## Abstract

The human visual system processes a massive amount of visual information very rapidly, requiring efficient coding mechanisms to handle such data within physiological constraints. The biological foundations of these mechanisms remain poorly understood. One hypothesis suggests that the visual system prioritizes the encoding of specific features of natural scenes optimized to maximize information transfer while minimizing computational costs (constrained-maximum entropy criteria). This study aims to identify a possible neural marker of this prioritizing mechanism. Participants were briefly shown stimuli with varying proportions of such *optimal* features, while EEG visual evoked response was recorded. Analysis focused on the C1 component, the earliest visual evoked component commonly considered to mainly reflect the first cortical response in the primary visual cortex (V1). Results revealed that the C1 component peaks earlier when elicited by *optimal* features, with a proportional speed-up following the gradual increase of their number. This provides evidence for an early efficient selection of optimally informative visual features in humans.

**Supplementary Information:**

The online version contains supplementary material available at 10.1038/s41598-025-07644-9.

## Introduction

Our visual system is constantly exposed to an overwhelming flux of information: every second, billions of photons are captured by our retina and transformed into representations of the world that we use to effectively interact with the environment^1^. The need for accurate representations is bounded by computational and metabolic limitations^2–4^ which impose the system to compress and code efficiently this massive amount of information as early as possible in the visual stream^5–7^. Despite their relevance for vision, the biological mechanisms of efficient coding are still largely unknown.

It has been proposed that vision, following constrained-maximum entropy criteria, prioritizes the encoding of specific features of natural images that maximize information transmission within given computational costs^8^. The constrained-maximum entropy principle, as applied to early vision, postulates that the visual system must optimize the amount of information preserved while respecting strict biological constraints such as limited processing capacity and metabolic efficiency. This approach assumes that the system must filter the incoming data stream, selecting only a subset of relevant features that are significant in natural scenes while discarding redundant or less informative features. By doing so, the system ensures that the most critical information is transmitted efficiently to higher-level processing areas without exceeding available resources. This process becomes particularly relevant when tight limitations on processing time are required. Following this optimization process, the features extracted are considered *optimal* features. According to this hypothesis, at an early stage, the visual system extracts a limited number of such *optimal* features to create simplified—but effective—representations of visual scenes (sketches^9,10^). All other features in the input that do not meet optimality criteria are discarded (*non-optimal* features).

In applying the model to vision, Del Viva et al.^8^ used a public database of 560 calibrated natural pictures^11^ (see examples in Supplementary Fig. 1a). Each image was digitized to 1-bit luminance (black/white) by setting the threshold at its median luminance value (Supplementary Fig. 1b). The need for such a strong reduction in levels is a corollary of the central idea of compression of the proposed pattern filtering model: indeed, the number of possible patterns—assumed to be a limited resource—increases exponentially with the number of allowed levels (that is, 2^n*N^, where *n* is the number of bits and *N* the number of pixels), as does the amount of computation needed to calculate them. Therefore, using a large number of levels in the model is not only impractical, but also defeats its very purpose of saving computational resources. For the same reason, the simplest possible set of features—defined as all possible configurations of 3 × 3 square pixel patches—was used. The probability distribution of all features in the set of digitized images was then evaluated, and the optimal set of patterns was extracted according to a constrained maximum entropy function (Supplementary Fig. 1c). The model has only two free parameters, corresponding to the two constraints considered: the number of patterns to be selected (N) and the output bandwidth of the system (W). According to the model, only the selected *optimal* features are used to filter images and create sketches (Supplementary Fig. 1d), while all other features—both those with high and low frequency of occurrence—are discarded.

The constrained-maximum entropy model, when applied to visual images, is then able to extract basic primitives such as edges and bars. These elements constitute the basis of edge detection, allowing the capture of important changes in properties of the world, which is one of the main goals of vision. Many early vision computational models aim to achieve the best performance in detecting object contours by using biologically plausible filters (edge- and bar-like shaped) based on a priori knowledge of physiological details^12–14^. The algorithm proposed by the reference model, instead, does not use prior knowledge about the properties of biological visual filters but derives them using optimality criteria. That is, the edge detection property of the visual system follows directly from very general principles, as the optimal solution for fast processing when dealing with an information bottleneck and limited computational resources^8^.

Past studies have shown that some known properties of early vision can be understood in terms of *efficient coding*, a theoretical framework proposing that sensory systems are optimized to reduce redundancy and maximize the transmission of informative components of natural stimuli^7,15,16^. Within this context, computational models such as *sparse coding* have demonstrated that optimizing for representational efficiency leads to the spontaneous emergence of edge- and orientation-selective units resembling V1 neurons^16–18^. Similarly, Independent Component Analysis (ICA) has shown that statistically independent components extracted from natural images give rise to receptive fields similar to those found in early visual cortex^19^. More recent developments include *predictive coding* approaches, where perception is framed as a process of minimizing prediction errors between expected and actual sensory inputs^20–22^. Additionally, models based on biologically plausible learning mechanisms, such as spike-timing-dependent plasticity (STDP), have also been shown to give rise to orientation and motion selectivity^23^.

Despite differences in assumptions and methodologies, these models share the common goal of explaining how selectivity to features like edges, orientations, and motion can emerge naturally from the statistical structure of the environment, without requiring strong a priori physiological assumptions. They converge on the idea that neural representations are shaped by principles of computational efficiency under ecological and resource constraints^24^. The model presented in this work aligns with this computational perspective but emphasizes lossy compression as a key optimization target^8^. Unlike sparse coding or ICA models, which aim for “almost-lossless” compression—often relying on a substantial number of free parameters^17^—our approach explicitly targets lossy compression. Our reference model indeed aims for strong information compression with no regard for the fidelity of image reproduction. Also, past computational models argued that the visual system devotes resources to detecting features of natural images in proportion to their probability of occurrence in the input^16,25^. Here, instead, discarding the most probable input configurations is necessary to fit within the output bandwidth limitations. Thus, the most common visual features, i.e., uniform luminance patches, are automatically rejected by the model as they occupy most of the transmission bandwidth and are inefficient to encode (see Supplementary Fig. 1c). This selection process naturally selects features of intermediate probability of occurrence, which are both informative and efficient to encode. Nonetheless, the features extracted (using only two free parameters), within the limitations of a 3 × 3 grid, are indeed functional representations of edges, bars, or L-shaped existing visual filters^26^ and provide a compressed representation effective for scene recognition^8^ (see Supplementary Fig. 1c).

This model has been supported by demonstrations that in “fast vision” —which refers to visual processing under strict temporal constraints (e.g., within 20–25 ms^27,28^—sensitivity is higher in response to the model-predicted *optimal* features than to the other features. Crucially, it was found that sensitivity saturates when images contain *optimal* features only, with no perceptual benefit by adding further features, suggesting that early visual representations mostly rely on these few *optimal* features^8^. More recent studies also show that *optimal* features attract the human gaze and attention and may interfere with saccade programming, further supporting their relevance in fast vision^29–32^. Overall, these findings support the existence of this constrained-maximum entropy, early-filtering mechanism in the human brain capable of rapidly and reliably processing the most informative pieces of information coming from the external world (i.e., this is what here we consider as “efficient coding”). Previous behavioral evidence also suggests that this mechanism may rely on the magnocellular pathway^33^, specialized in fast and coarse vision^34,35^.

This work aims to provide a clear neurophysiological fingerprint of this efficient coding mechanism to expose its neural substrates. To this purpose, we recorded the earliest EEG cortical component evoked by a visual stimulus, known in the literature as the C1 component^36^.

The C1 component originates in the striate cortex and mainly reflects a feedforward response to visual stimuli, with a typical onset latency ranging from 40 to 70 ms, peaking around 60–100 ms from stimulus onset^37^. This component exhibits a characteristic polarity reversal, reflecting the retinotopic organization of V1, for which upper visual-field stimulation elicits a negative C1 and vice versa^36^. Despite being primarily considered an exogenous component, both low-level and high-level factors can modulate—but differently—its characteristics: for instance, changes in stimulus size, contrast, and shape are known to modulate C1 latency and amplitude^38,39^, while selective attention^40^ and perceptual learning^41^ have been demonstrated to affect its amplitude.

Given that the extraction of *optimal* features is assumed to occur at the earliest stage of analysis, one expects an earlier cortical processing for these features than for *non-optimal*, which should be reflected in a faster C1 component response.

## Results and discussions

**Fig. 1:**
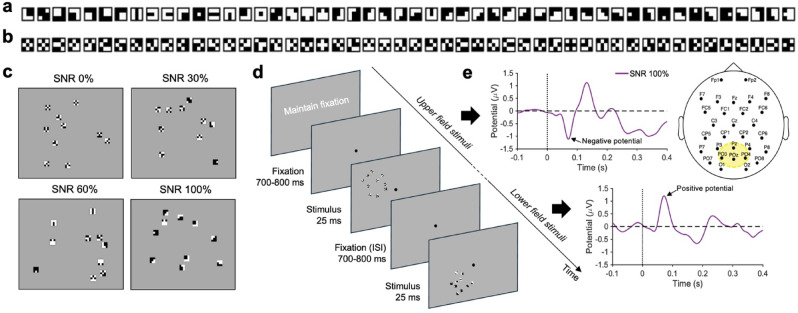
Methods outline. (**a**) *Optimal* features. Set of black and white features selected by the reference constrained-maximum entropy model^8^. (**b**) *Non-optimal* features. Set of black-and-white features discarded by the reference model^8^—i.e., those with the lowest probability of occurrence in the statistical distribution of all possible features. (**c**) Examples of stimuli SNR. Stimuli are composed of ten tiny features (0.3°) randomly positioned in an ideal compound of 3° diameter, without overlapped positions (mean distance between features = 0.3°). (**d**) Task. Trials started with the presentation of a fixation point in the center, followed by the presentation of one stimulus (eccentricity from central fixation = 4°), keeping the fixation point visible in the center of the screen. Stimulus positions were located at polar angles of 25° above and 45° below the horizontal meridian in the upper- and lower-left visual field, respectively^37^. (**e**) Data processing. Grand-average VEP waveform, for SNR 100% condition, from the electrode cluster shown in the scalp outline. When the stimulus was displayed in the upper/lower field, it—respectively—evoked an early negative/positive response (C1 component, upper/lower traces), peaking at around 70–80 ms from stimulus onset. VEPs were computed over a set of electrodes a-priori determined (POz, PO3, PO4, Pz; see the yellow area in the head model).

To test the hypothesis that the visual system prioritizes the encoding of *optimal* visual features from the earliest stages of cortical processing, we recorded the C1 component in response to visual stimuli that gradually varied in their feature optimality. Stimuli consisted of compounds composed of ten features, randomly selected from two sets of *optimal* and *non-optimal* features, each containing 50 features (Fig. 1a,b, respectively) as previously calculated by Del Viva et al.^8^. Among all possible discarded, *non-optimal* features, we specifically selected the 50 with the lowest probability of occurrence within the statistical distribution of all 512 possible features (see Supplementary Fig. 1c). This selection ensured that the two sets were matched in number and separated by a significant gap in terms of probability of occurrence. The stimulus compounds were constructed to vary in their ratio of *optimal* to *non-optimal* features—a ratio we refer to as the 'signal-to-noise ratio’ (SNR), with levels of 0%, 30%, 60%, and 100% (Fig. 1c), where, for example, an SNR of 30% corresponds to 3 *optimal* and 7 *non-optimal* features.

In the majority of the trials, participants were asked to fixate a point displayed on a monitor, while stimuli were quickly presented (25 ms), either in the upper or lower visual field (Fig. 1d). To isolate the C1 component—for each participant and stimulus type—we subtracted the response evoked from the upper and lower visual field stimulation (Fig. 1e). Because of the anatomical organization of the primary visual cortex, the dipoles for the upper and lower fields point in opposite directions, resulting in opposite response polarities^36,42^. Subtracting the two VEPs thus highlights activity reflecting the typical polarity reversal of the component, while evoked activity that does not systematically differ between upper and lower visual field stimulation is canceled out^43^. Computing the upper-lower difference VEP waves led to the exclusion of 9 participants who did not exhibit a detectable C1 component across all SNRs (see *Methods* section for further details). This proportion of exclusions likely results from individual anatomical variability, particularly in the orientation of the calcarine sulcus^44^, and from differences in signal-to-noise ratio that disproportionately affect early components like the C1^37^.

**Fig. 2:**
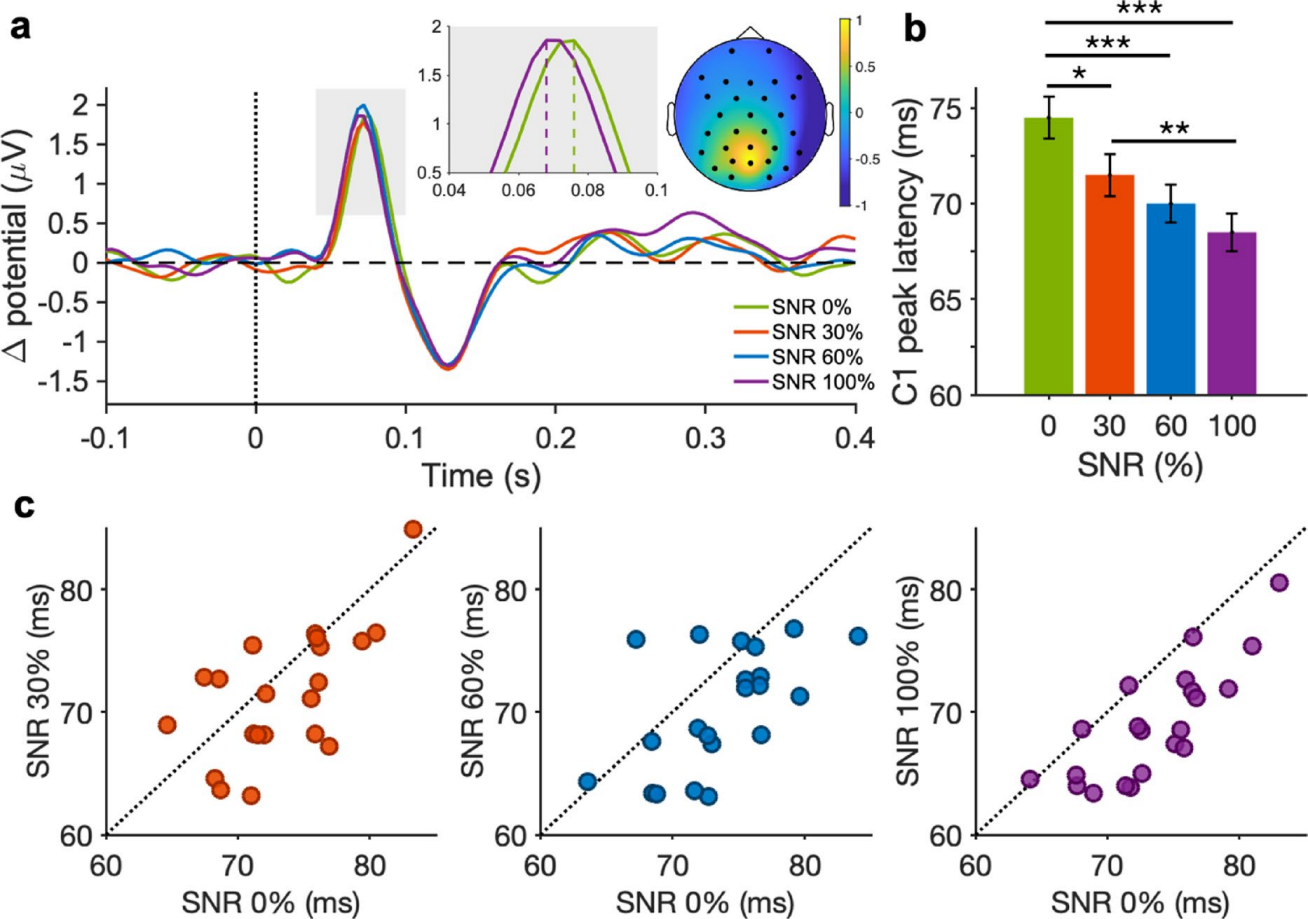
C1 component results. (**a**) Average upper-lower difference VEP waves across different SNR conditions. The inset shows a zoomed-in view of the VEP waves at the C1 peak latencies for SNR 0% and 100%, and the scalp topography at the time of its peak. (**b**) C1 peak latency as a function of SNR levels. Post-hoc t-tests (Bonferroni correction) show a significant difference between latencies of stimuli with SNR 0% vs. 30% (*t* = 2.79, *p* < 0.05, *d* = 0.43—*moderate* effect size, 95% CI [0.47, 0.92]), SNR 0% vs. 60% (*t* = 4.31, *p* < 0.001, *d* = 0.67—*large* effect size, 95% CI [0.14, 1.2]), SNR 0% vs. 100% (*t* = 6.08, *p* < 0.001, *d* = 0.94—*large* effect size, 95% CI [0.34, 1.56]), and SNR 30% vs. 100% (*t* = 3.29, *p* < 0.01, *d* = 0.51—*moderate* effect size, 95% CI [0.17, 1.009]). Asterisks mark statistically significant pairwise comparisons across SNR conditions: * p < 0.05, ** p < 0.01, *** p < 0.001. Error bars are s.e.m. across participants. (**c**) Individual observer’s comparisons of C1 peak latencies between SNR 0% and higher SNR levels. Each dot represents a single participant. Dashed line marks the equality line.

Figure 2a shows upper-lower difference VEP waves, averaged across participants (n = 21), for the different SNR conditions. All traces clearly show a prominent positive component expressed over the occipital and parieto-occipital electrodes, peaking around 70 ms after stimulus onset, thus reflecting the C1 component. Once the C1 was detected, we computed its peak latency, onset, amplitude, prominence, and width (see Supplementary Fig. 2 for a graphical illustration of all the parameters extracted). The inset displays the VEP waves at the C1 peak latencies for the two extreme SNR conditions (0% and 100%), highlighting a difference in their latency, with an earlier C1 latency in response to *optimal* (SNR 100%) as compared to *non-optimal* features (SNR 0%). To quantify the link between visual features and C1 response, we estimated the C1 peak latency for each individual and SNR condition. Notably, C1 peak latency decreased with increasing the proportion of *optimal* features in the stimulus compound (Fig. 2b; C1 peak latency for SNR 0%: 0.074 ± 0.001 s; SNR 30%: 0.071 ± 0.001 s; SNR 60%: 0.070 ± 0.001 s; SNR 100%: 0.069 ± 0.001 s. Mean ± s.e.m.). One-Way ANOVA confirmed the presence of a significant main effect of SNR on C1 latency (F (3, 60) = 13.28, *p* < 0.001, η^2^ = 0.39—*large* effect size; pairwise comparisons *t*-tests across different SNR conditions are reported in the caption of Fig. 2). On average, higher SNR stimuli resulted in shorter C1 peak latencies with a maximal difference of 5 ms (over about 70 ms) between processing only *optimal* or only *non-optimal* features. Figure 2c displays individual C1 peak latency data for all participants, comparing 0% SNR stimuli with stimuli at higher SNR levels; for almost all participants, the C1 latency decreases as SNR increases, with the shortest latency observed for 100% SNR stimuli. See Supplementary Fig. 3 for checking the C1 peak latency of the nine excluded participants, in the SNR condition(s) where C1 was detected.

Beyond peak latency, peak onset is another informative temporal marker^45^. Mirroring the effects found for peak latency, C1 onset changes as a function of SNR (see Supplementary Fig. 4). All other parameters of the C1 component, i.e., peak amplitude, prominence, and width, did not differ across SNR conditions (Supplementary Fig. 4). Therefore, measured latencies are not affected by curve widths that, being similar, allow clear peak segregation (see Fig. 2a—inset). Since *optimal* and *non-optimal* features in the stimuli compound were matched for low-level features like luminance, contrast, and size, the resulting neural responses likely had similar amplitudes^38^.

As additional analysis, we computed VEP waves including only the trials in which stimuli were presented in the upper visual field; results are consistent with the upper-lower difference analysis (see Supplementary Fig. 5). Instead, analyzing only the lower-field response tends to conflate the C1 with overlapping components such as P1, which undermines the spatial specificity and temporal resolution required to isolate our targeted component, making this analysis uninformative on its own.

VEP waves in Fig. 2a show a visible C2 component, which is hypothesized to reflect feedback from higher visual areas to the early visual cortex^43^. As a control, we also measured the properties of this component, finding that any of the C2 parameters—peak latency, amplitude, prominence, and width—differ across SNR conditions (see Supplementary Fig. 6).

**Fig. 3:**
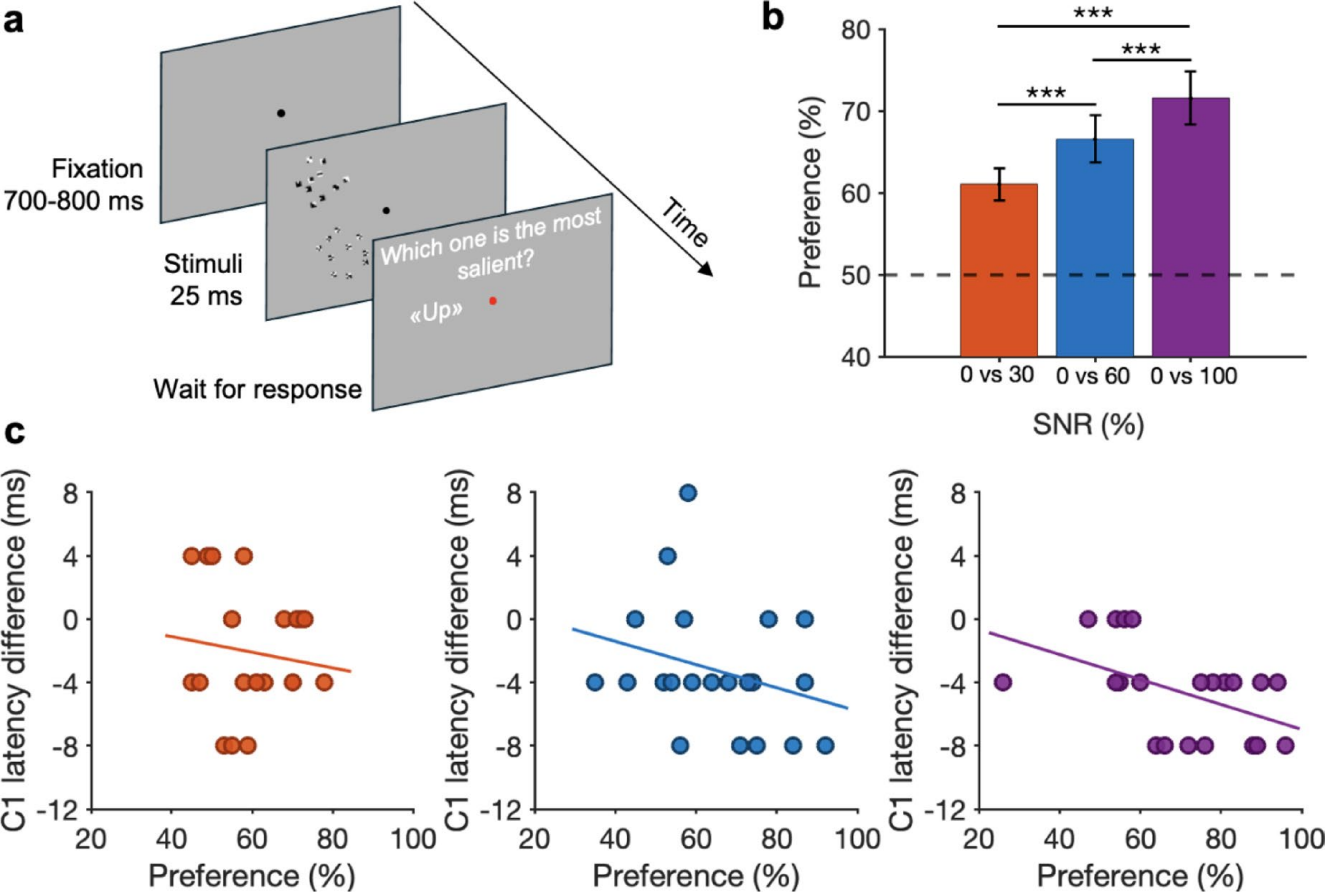
Preference for *optimal* features. (**a**) Behavioral task. Two compounds were simultaneously presented in the upper and lower visual field, one containing only *non-optimal* features (SNR 0%) and the other containing a variable number of *optimal* features (SNR 30%, 60%, 100%). In this example participants had to choose the most salient stimulus between SNR 100% (up) and SNR 0% (down)—see Fig. 1C for examples of stimuli with different SNR. (**b**) Average preference (N = 30) for SNR 30%, 60%, and 100% against SNR 0%. One-Way ANOVA analysis confirms the presence of a significant main effect of SNR on preference (F (2, 58) = 26.89, *p* < 0.001, η^2^ = 0.48—*large* effect size). Pairwise comparisons *t*-tests show significant differences across SNR conditions: 30% vs 60% (*t* = -3.84, *p* < 0.001, *d* = − 0.366—*small* effect size, 95% CI [− 0.634, − 0.097]), 30% vs 100% (*t* = -7.33, *p* < 0.001, *d* = − 0.698—*medium* effect size, 95% CI [− 1.030, − 0.366]), and 60% vs 100% (*t* = -3.49, *p* < 0.001, *d* = − 0.332—*small* effect size, 95% CI [− 0.596, − 0.069]). (**c**) Correlations between individual preference for *optimal* features and C1 peak latency differences between different SNRs. Left panel—SNR 30% vs 0%. r = − 0.07, *p* = 0.75. Middle panel—SNR 60% vs 0%. r = − 0.28, *p* = 0.21. Right panel—SNR 100% vs 0%. r = 0.051, *p* < 05.

In a small percentage of trials, unpredictably, two compounds of features were simultaneously presented both in the upper and lower visual field, one containing only *non-optimal* features (SNR 0%) and the other containing a variable number of *optimal* features (SNR 30%, 60%, 100%) (see Fig. 3a). In these trials, participants were asked to choose “*the most salient stimulus*” (2AFC task). These trials were discarded from the EEG analysis. This behavioral task was introduced to measure the individual preference for *optimal* vs. *non-optimal* features as a function of SNR. Confirming previous findings^32^, we indeed found a reliable and strong preference for *optimal* features, even at low SNR (N = 30; see Fig. 3b). As a control, we computed the behavioral preference of the excluded participants only. Their average preference (N = 9) for SNR 30%, 60%, and 100% against SNR 0% resulted to be 63 ± 5%, 70 ± 6%, and 76 ± 6% (Mean ± s.e.m.), respectively. Consistent with the overall sample, these participants thus show increasing preference as a function of SNR. This indicates that the absence of a prominent C1 in one or more stimulation conditions cannot be attributed to a lack of behavioral preference for *optimal* features.

Observers’ preference was then correlated with the EEG responses to test whether higher individual preference is correlated with shorter C1 peak latency (N = 21; Fig. 3c). Results showed a trend for which higher preference for SNR 30%, 60%, and 100% vs. SNR 0% positively correlate with the latency difference between those SNR pairs (correlation reaches significant threshold for SNR 100% vs 0%; all Spearman’s correlation results are reported in the caption of Fig. 3). Therefore, subjective explicit preference for *optimal* features seems to be related to the EEG responses to the stimuli; the higher the preference, the shorter the C1 latency.

This work thus leads to a concise, yet to-the-point conclusion: *optimal* features are processed faster than *non-optimal* features. Moreover, prioritization of *optimal* feature encoding seems to be related to their higher perceived saliency.

Achieving this result was possible because the reference model provides a precise recipe for differentiating between visual features used in the data compression process and those that are not. Notably, the *non-optimal* features used in this study—being the least frequent in the statistical distribution of natural image features—individually convey high information content. However, precisely because of their rarity, a large number of such features would be required to achieve an efficient representation of a visual scene. This makes them “non-optimal” in contexts where compression is necessary. Thus, the evidence that these specific *non-optimal* features are processed more slowly than the *optimal* ones further supports the role of computational constraints in shaping the prioritization of visual feature processing.

The choice of this particular set of *non-optimal* features was motivated by two main reasons. First, it allowed us to extract 50 features, thus matching the number of *optimal* features and enabling balanced and randomized stimulus construction—each stimulus being composed of 10 features. Second, this set is markedly separated in terms of probability of occurrence from that of the *optimal* features. This would not have been feasible using features from the opposite end of the statistical distribution (i.e., the most frequent ones), as they are not only few in number but also very close in occurrence probability to the *optimal* features, preventing a clear separation between the two sets. While methodologically justified, this selection may nonetheless represent a limitation of the present study. Future works could aim to compare *optimal* features with other types of *non-optimal* features drawn from different regions of the probability distribution—such as the most common ones.

An important consideration regarding the spatial frequency content of our stimuli deserves discussion. In our task, each compound stimulus was created trial-by-trial by randomly selecting ten features from predefined sets of *optimal* and *non-optimal* features. These features were not selected based on their spatial frequency content, but rather on their probability of occurrence within natural images (see Supplementary Fig. 1c). Nonetheless, we performed a-posteriori analyses to investigate the spatial frequency properties of the stimuli presented to participants. Specifically, we computed the power spectral density of all stimuli and compared their spatial frequency distributions across SNR levels (0%, 30%, 60%, and 100%) (see Supplementary Figure S7). While all stimuli spanned the same overall range of spatial frequencies, from 0 to 12 cycles per degree, those with a higher proportion of *optimal* features exhibited greater power at lower spatial frequencies. Notably, the model does not incorporate any a priori physiological knowledge about the anatomical organization of V1 neurons (e.g., edge- and bar-like receptive fields), nor does it explicitly consider the existence of distinct classes of neurons with different spatio-temporal frequency tuning (i.e., magnocellular and parvocellular). Therefore, we argue that general principles of constrained information maximization led to the selection of low spatial frequency features that align with those processed faster in the early stages of vision^46^—an outcome that underscores the model’s predictive power. This could imply that the statistical structure of natural images, coupled with information efficiency constraints and computational limitations, may have played a role in shaping the key characteristics of early visual processing, providing an interesting perspective on why V1 neurons may favor specific receptive field structures. Further studies are necessary to disentangle the contribution of spatial frequency and probability of occurrence on C1 latency, for example, by specifically creating stimuli with different degrees of optimality but equal spatial frequency content.

Considering the source of the C1 component, it is reasonable to suggest that this efficient encoding mechanism operates in the early stages of processing. Due to its characteristic polarity reversal, the C1 is traditionally associated with V1^36^; however, contributions to C1 from extrastriate areas such as V2 and V3 cannot be excluded^44,47–49^.

On a more general ground, the present findings, joined with previous works^29–33^, lead us to speculate that the efficient mechanism proposed here is in charge of the creation of bottom-up saliency maps of visual scenes. That is, we propose that these maps are created by quickly extracting a few specific optimally informative, salient features—those that best balance information gain and computational efficiency. V1 appears to be the most likely neural substrate for these maps. This hypothesis is also corroborated by the observations related to the fast response timing of the primary visual cortex’s neurons^50,51^ and the similarity of their elongated receptive fields^26^ with the spatial structure of predicted *optimal* features^8^. Orientation sensitivity is indeed a response property typically associated with visual cortical cells in mammals, and there is substantial evidence in favor of Hubel and Wiesel’s model of orientation-selectivity (OS) emerging from selective wiring from LGN to V1^26^. However, growing evidence shows OS responses in mice and primates also in earlier structures such as the LGN^52–55^, SC^56–58^, and the retina^59,60^. However, we still argue that V1 can be the neural correlate of the proposed bottleneck mechanism because V1 is the most extended visual area, with intensive energy consumption, and a high input/output neural ratio^2,61^. This hypothesis also aligns with previous proposals that V1 acts as a pre-attentive filter, performing a lossy pre-attentive selection of information^62,63^, similar to the mechanism implemented by our model^8^.

Additionally, the absence of sensitivity to stimulus optimality in C2 signals suggests that the observed response to *optimal* features probably does not depend on feedback from extrastriate areas, more likely reflecting the activity of feedforward bottom-up saliency mechanisms.

We finally speculate that this early compressive stage in our brain not only determines behavioral saliency but also acts as the “gatekeeper” of visual perception, defining what information passed to subsequent stages of analysis.

Finally, it’s worth discussing the implementation of the proposed algorithm on dedicated Deep Neural Networks models (DNN), heavily used nowadays to understand human vision^64–66^. The parallelization of our algorithm into a DNN made of autoencoders (AEs)^67^ would be interesting, for example, for implementation reasons. Indeed, the algorithm in question bears some resemblance to AEs, as it transforms a set of high-dimensional input data into a compressed and efficient representation using a specific encoding function. It is certainly conceivable that an AE incorporating maximum information principles^68^ could converge to the same results found with the constrained maximum-entropy method. Such a calculation could be developed a posteriori, starting from the proposed solution as a target, which would make it much easier to obtain. We do have a work in progress on the implementation of this model on dedicated field-programmable gate arrays (FPGAs), which would allow for the rapid processing of large volumes of natural images and for extending the framework to dynamic inputs, potentially enhancing representational bit depth. However, we believe that the interest of an NN-based implementation lies mostly in the practical aspect of computational convenience and in biological plausibility, which the authors purposely excluded in formulating the algorithm, to concentrate purely on its abstract optimality^8^. The observation that the human visual system actually seems to follow such feature selection rules is certainly an encouragement in pursuing future work in the search for possible biologically plausible implementation mechanisms.

## Conclusions

This study addresses a fundamental issue: the visual system must efficiently analyze the visual scene within a short time frame to ensure survival. Here we explored the notion that this is done through a mechanism capable of rapidly and reliably processing the most informative pieces of information coming from the external world. Indeed, our results suggest the existence of an early “gatekeeper” mechanism in early visual stages, probably V1, capable of prioritizing the most salient features of a visual scene. The findings of this study are a step forward in bridging the gap between the computational nature of the constrained-maximum entropy model and its biological, neural implementation. This work motivates further research to refine its neural description and extend its application to more natural, dynamic inputs.

## Materials and methods

### Participants

A total of 30 volunteers (23 females, 7 males; mean age = 26 ± 3.1 s.d.) participated in the study. All participants had normal or corrected-to-normal vision and did not present any neurological or psychiatric disorders that could influence neural functioning. The experimental procedure is in line with the Declaration of Helsinki and follows the ethical procedures approved by the Local Research Subjects Review Board (“Commissione per l’Etica della Ricerca”, University of Florence, 23rd September 2021, n. 174). Informed consent was obtained from all participants.

### Apparatus

Measurements were conducted in a quiet room under dim lighting conditions. The stimuli were generated using MATLAB (R2016b, MathWorks) with Psychtoolbox extensions^69–71^ and displayed on a gamma-calibrated AOC gaming 27G2U/BK monitor (resolution: 1920 × 1080 pixels; refresh rate: 120 Hz). The monitor was positioned 57 cm in front of the observer, subtending a visual angle of 61 × 34°.

Electrophysiological (EEG) activity was continuously recorded at 500 Hz using a 10/20 g. Tech system (g. Nautilus Multi-Purpose system). EEG was recorded from 30 active channels: FP1, FP2, F7, F3, Fz, F4, F8, FC5, FC1, FC2, FC6, C3, Cz, C4, CP5, CP1, CP2, CP6, P7, P3, Pz, P4, P8, PO7, PO3, POz, PO4, PO8, O1, O2, referenced to the right earlobe. The electrode impedance, for each electrode, was checked at the beginning of each session and kept below 30 k$\omega$. The synchronization between the EEG activity and visual stimulation was assured by means of a photocell triggering the onset of the stimulation period to the EEG recording.

### Stimuli

Stimuli were arrays of ten different features, arranged in an ideal circle (radius 1.5°) subtending 3° of visual angle (diameter 3 cm). Each single feature in the stimulus subtended 0.3 × 0.3° (9 × 9 pixel), and the minimal distance between features was 0.3° (i.e., no overlapped positions).

The features were extracted from images from the McGill Calibrated Color Image Database^11^, digitized at one bit, and subdivided into 3 × 3 pixel features. The entropy yield per unit cost was then calculated for each feature (512 in total), based on presentation frequency and required computational energy—i.e., applying the constrained-maximum entropy function^8^ (see Supplementary Fig. 1). The *optimal* features set was chosen by setting a maximum number of 50 features with a maximum output bandwidth occupancy of 0.025^8^ (see Fig. 1a). A set of *non-optimal* features was also chosen, specifically containing the 50 features with the lowest probability of occurrence, that is those fulfilling a maximum entropy but not a constrained-maximum entropy criterion (see Fig. 1b). The two sets of features do not differ, on average, in their luminance contrast properties (*optimal* features—average greyscale value = 129 ± 51 (s.d.); Weber contrast = 0.016; RMS contrast: M = 1.5, s.d. = 0.002, range [1.495 1.505]; *non-optimal* features—average greyscale value = 127 ± 22 (s.d.), Weber contrast = 0; RMS contrast: M = 1.5, s.d. = 0.001, range [1.498 1.502]). An independent *t*-test on the greyscale values of the optimal and non-optimal features was conducted to verify that the luminance difference was negligible, revealing no significant difference (t(98) = − 0.43, *p* = 0.67). Spatial frequencies of the two sets are also largely overlapped: the theoretical range of the unidimensional spatial frequency for our 9 × 9 pixels features is between 3.84 and 11.52 cycles/deg for both sets (pixel angle = 0.0289 deg) with *non-optimal* features containing on average more of the higher spatial frequencies. However, the distance of the spatial frequency spectra within and between the two sets of features do not differ by much more than the typical distances within each individual set, and they both extend over essentially the whole frequency space theoretically allowed for their size^29^.

Features in the stimulus compound were randomly selected with replacement (at each trial) from the two sets. The compounds created could contain 0, 3, 6, or 10 *optimal* features. This subdivision allowed us to present the participants with stimuli containing four different signal-to-noise ratios (SNR): 0%, 30%, 60%, 100% (see Fig. 1c).

For stimuli’ spatial frequency analysis, all SNR stimuli presented to all participants were considered. The power spectrum density was obtained by calculating a two-dimensional Fourier Transform (2D-FFT) on a central square region of 1080 × 1080 pixels. Then, the radial spectra were calculated on each image, and subsequently, the spectra were averaged to obtain the radial average power spectral density. Average spatial frequency spectra distributions of all SNR stimuli were compared using Kolmogorov–Smirnov tests (see caption of Supplementary Fig. 7 for results).

### Procedure

At the beginning of the experiment, participants read the instructions on the screen. As soon as they were ready, the experiment started. Stimuli were flashed for 25 ms duration against a gray background (44 cd/m^2^) that was isoluminant with the mean luminance of the features (black = 3 cd/m^2^, white = 90 cd/m^2^).

A total of 2280 trials were run, divided into 6 blocks of 360 trials to avoid attentional loss. In the majority of the trials (1920 over 2280), stimuli were briefly presented binocularly one at a time (25 ms) in randomized sequences to the two quadrants of the left visual field at a fast rate (Inter-stimulus-interval—ISI, varying between 700 and 800 ms). During ISI a small black fixation point was shown to guide observers in maintaining fixation (see Fig. 1d). In a few trials (360 over 2280), a Two-Alternative Forced Choice task (2AFC) was introduced. In this portion of the trials, participants were shown two stimuli at the same time and were asked to choose the one that they considered “more salient” by pressing up or down on the keyboard as quickly as possible. Stimuli still disappeared after 24 ms, and a red fixation point remained on the screen until response (see Fig. 2a).

In all trials, stimulus positions were centered along an arc that was equidistant (4°) from a central fixation point and located at polar angles of 25° above and 45° below the horizontal meridian. These asymmetrical positions were chosen so that the upper and lower field stimuli would stimulate approximately the opposite zones of the lower and upper banks of the calcarine fissure, respectively. This choice is based on the findings that the horizontal meridian is actually represented on the lower bank rather than at the lateral recess of the calcarine fissure^36,72^.

### Data processing and statistical analysis

#### Behavioral data

For each participant (N = 30), we calculated the preference for *optimal* features as a function of SNR obtained in the trials requiring a behavioral choice (2AFC), i.e. how many times, in the trials showing two arrays, observers choose those containing more *optimal* features. Preferences distributions resulted to be normally distributed (Shapiro–Wilk test yielded* p* > 0.05); parametric statistical tests were then used. Specifically, average preference for SNR 30%, 60%, and 100% against SNR 0% were compared with a One-Way repeated measure ANOVA. Effect sizes were estimated by eta-squared statistics (*η2*). Pairwise comparisons were done with post hoc Student’s t-tests with Bonferroni corrections. The effect size of differences between conditions was estimated by Cohen’s *d* statistics with 95% confidence intervals.

#### EEG preprocessing

EEG analyses were performed with the MATLAB toolbox EEGLAB (r2016b, MathWorks^73^, FieldTrip^74^, and custom codes. The signal was high-pass filtered (cut-off of 0.1 Hz, Blackman sinc FIR filter with a transition bandwidth of 0.2 Hz), a Notch filter was applied to remove line noise (cut-off between 49 and 51 Hz, Blackman sinc FIR filter with a transition bandwidth of 0.2 Hz), and the EEG was re-referenced to a common average. Finally, EEG data were epoched around the time of stimulus onset. Baseline activity was defined as the average activity in the 150 ms before stimulus onset. A 4th-order zero-phase Butterworth IIR low-pass (35 Hz) was applied to the visual evoked potentials (VEPs). Main analyses, after discarding trials with periods of missing signal (1.33% of trials), were based—for each participant and condition—on 237 ± 3 trials (mean ± 1 s.d.).

#### C1 component

The VEP was first independently computed for stimuli presented in the upper or lower visual field, utilizing a-priori defined parietal and parieto-occipital electrodes (POz, PO3, PO4, Pz; see the head model in Fig. 1e). Electrodes’ selection was based on prior studies demonstrating that these sites best capture early visual activity originating from V1^37,42^. To isolate the C1 component, the responses evoked by stimulation in the lower and upper visual field were then subtracted^43^. The C1 peak latency, onset, amplitude, prominence and width, was determined, for the resultant VEP difference (lower minus upper field), as the local positive peak occurring between 40 and 100 ms, with a peak prominence and height exceeding 0.2 $\upmu$V (see Supplementary Fig. 2). Visual inspection was finally performed to verify the outcome of the automated data processing and remove any trial still showing important deviations from the mean (749 trials over 57,600—i.e., 1.3% of the total—were removed).

Computing the upper-lower difference VEP waves led to the exclusion of 9 participants; although all exhibited a prominent C1 in at least one SNR condition (four showed a prominent C1 response in 3 out of 4 SNR conditions; three showed C1 in 2 out of 4 conditions; and two showed a C1 response in just one SNR condition—see Supplementary Fig. 3), missing values in any condition prevented their inclusion in the within-subjects analysis performed across the four SNR conditions.

Statistical analyses on the C1 component parameters were then conducted on the remaining 21 participants. Because C1 parameters (latency, onset, amplitude, prominence, and width) distributions resulted to be normally distributed (Shapiro-Wilks test yielded *p* > 0.05), parametric statistical tests were used. Specifically, One-Way repeated measure ANOVAs were formed to compare means of C1 parameters across different SNR conditions. Effect sizes were estimated by eta-squared statistics (*η2*). Pairwise comparisons were done with post hoc Student’s t-tests with Bonferroni corrections. The effect size of differences between conditions was estimated by Cohen’s *d* statistics with 95% confidence intervals.

Additionally, the same statistical analyses were applied to the C1 peak latency, amplitude, prominence and width, extracted from VEP waves including only the trials in which stimuli were presented in the upper visual field (N = 21).

As a control, we also detected the immediate subsequent component following C1, namely the C2 component^43^, as the local negative peak occurring between 90 and 180 ms. The same statistical analyses (N = 17) described above were applied to the C2 peak latency, amplitude, prominence, and width across different SNRs.

Finally, individual observers’ preferences for *optimal* features—obtained between SNR 30%, 60%, and 100% against SNR 0%—were correlated with the C1 peak latency differences between those three SNR pairs (Spearman’s correlation).

## Electronic supplementary material

Below is the link to the electronic supplementary material.

Supplementary Material 1

## Data Availability

All data are available from the Zenodo database: 10.5281/zenodo.15025260.
