# Supplementary material for: Optimal feature encoding in early vision
Source: Sci Rep. 2025 Jul 3;15:23794. doi: 10.1038/s41598-025-07644-9 (PMC12229605; doi:10.1038/s41598-025-07644-9)
Supplement: Supplementary file 1 — Supplementary Material 1 [file 41598_2025_7644_MOESM1_ESM.pdf]

Supplementary Material for
"Optimal Feature Encoding in Early Vision"

Serena Castellotti, Giacomo Mazzotta, Alessandro Benedetto, & Maria Michela Del Viva

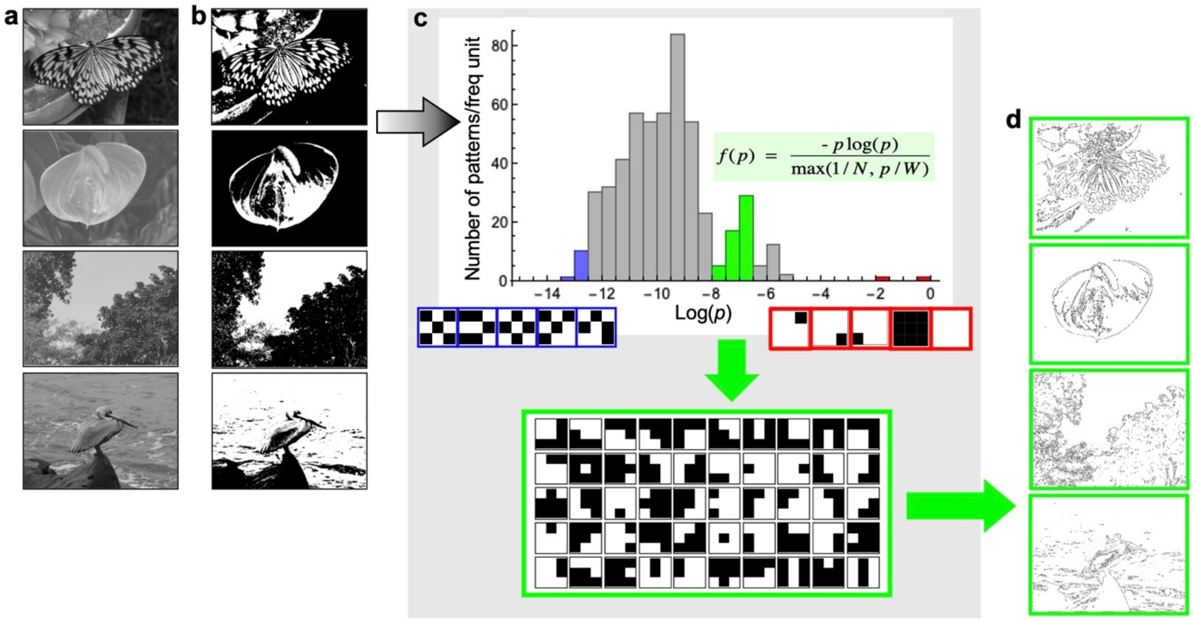

Supplementary Figure 1. Constrained maximum-entropy model. (a) Examples of images used (768x576 pixels). **(b)** 1-bit black/white digitized versions of images in (a). **(c)** Probability distribution of all 512 black and white 3X3 pixel patches (features) as a function of their probability of occurrence in the image database (*p*). In green the selected features that maximize the constrained maximum-entropy function (see equation) by using N=50 and W=0.025 (*optimal* features). Examples of low (blue) and high (red) probability features discarded by the model. **(d)** Sketches obtained from the images in (b) by retaining only the selected optimal features (green in (c)).

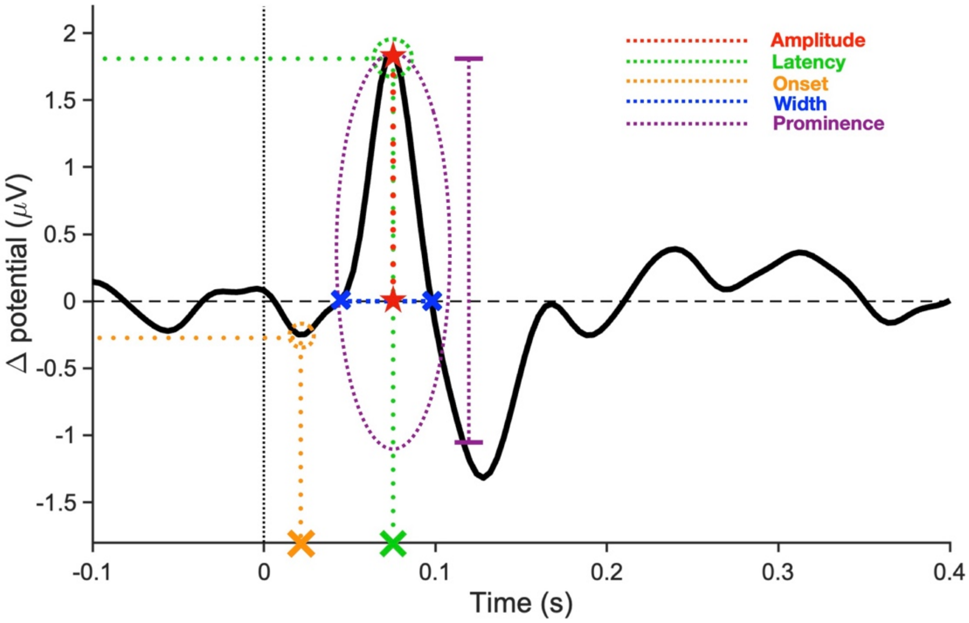

Supplementary Figure 2. C1 component parameters. To isolate the C1 component of the visual evoked potential (VEP), we adopted a differential approach by subtracting EEG responses to stimuli presented in the upper and lower visual fields along the horizontal meridian. This method enhances the visibility of the polarity inversion characteristic of the C1 component, typically originating in the primary visual cortex (V1). The C1 peak was identified using MATLAB's findpeaks function within a latency window of 40–100 ms post-stimulus. C1 onset latency was defined as the first time point preceding the peak at which the waveform crossed 50% of the peak amplitude[1]. C1 peak latency refers to the time point at which the maximum deflection (positive in the difference waveform) occurs within the predefined window. C1 peak amplitude is measured as the voltage difference between the pre-stimulus baseline and the peak. C1 peak prominence is defined as the vertical distance between the peak and the highest of the two valleys (or edges) that delimit the peak's boundary, quantifying how much the peak stands out from surrounding activity. C1 peak width was calculated as the full width at half maximum (FWHM), indicating the duration between the points on either side of the peak where the signal reaches half its maximum amplitude. For additional details on the peak detection method, see: https://www.mathworks.com/help/signal/ref/findpeaks.html

[1] *Kappenman, E. S., & Luck, S. J. (2011). ERP components: The ups and downs of brainwave recordings. In Oxford University Press eBooks. https://doi.org/10.1093/oxfordhb/9780195374148.013.0014*

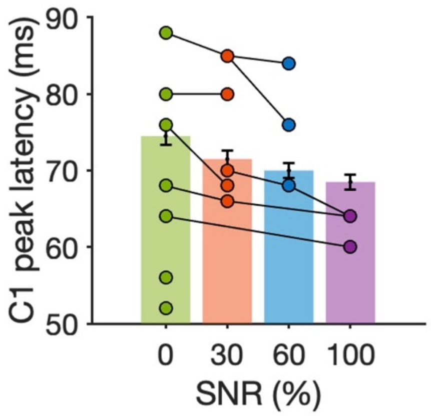

Supplementary Figure 3. C1 peak latency as a function of SNR levels. Individual data points from excluded participants (N=9), in the SNR condition(s) where C1 was detected, are shown overlaid on the average data (N=21) presented in Figure 2b. C1 peak latency trend across SNR levels is consistent with the main result, despite the presence of missing values.

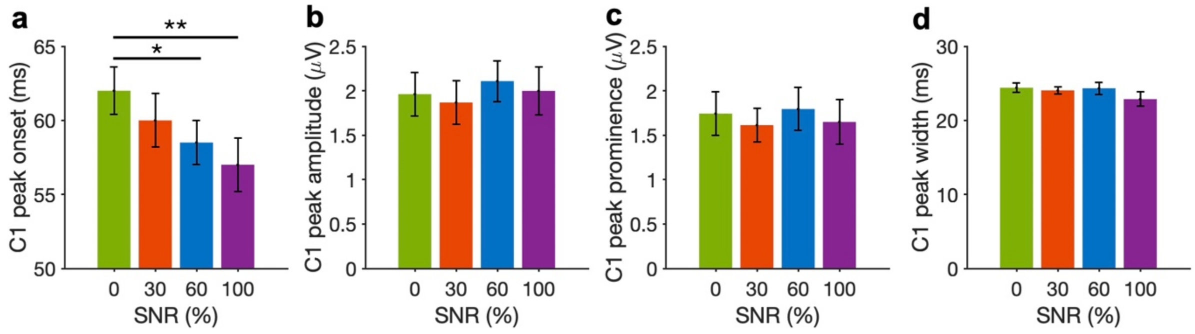

Supplementary Figure 4. Additional C1 component parameters. (a) C1 onset as a function of SNR levels (F (3, 60) = 4.53, $p < 0.01$, $\eta^2 = 0.185$ – *large* effect size). Post-hoc t-tests (Bonferroni correction) show a significant difference between latencies of stimuli with SNR 0% vs. 60% ($t = 2.78$, $p < 0.05$, $d = 0.62$ – medium effect size, 95% CI [-0.05 1.3]), SNR 0% vs. 100% ($t = 3.44$, $p < 0.01$, $d = 0.78$ – medium effect size, 95% CI [0.07, 1.47]. Asterisks mark statistically significant pairwise comparisons across SNR conditions: *p < 0.05, **p < 0.01. **(b)** C1 peak amplitude as a function of SNR levels (F (3, 60) = 0.64, $p = 0.6$). **(c)** C1 peak prominence as a function of SNR levels (F (3, 60) = 0.8, $p = 0.5$). **(d)** C1 peak width as a function of SNR levels (F (3, 60) = 2.04, $p = 0.12$). Error bars are s.e.m. across participants.

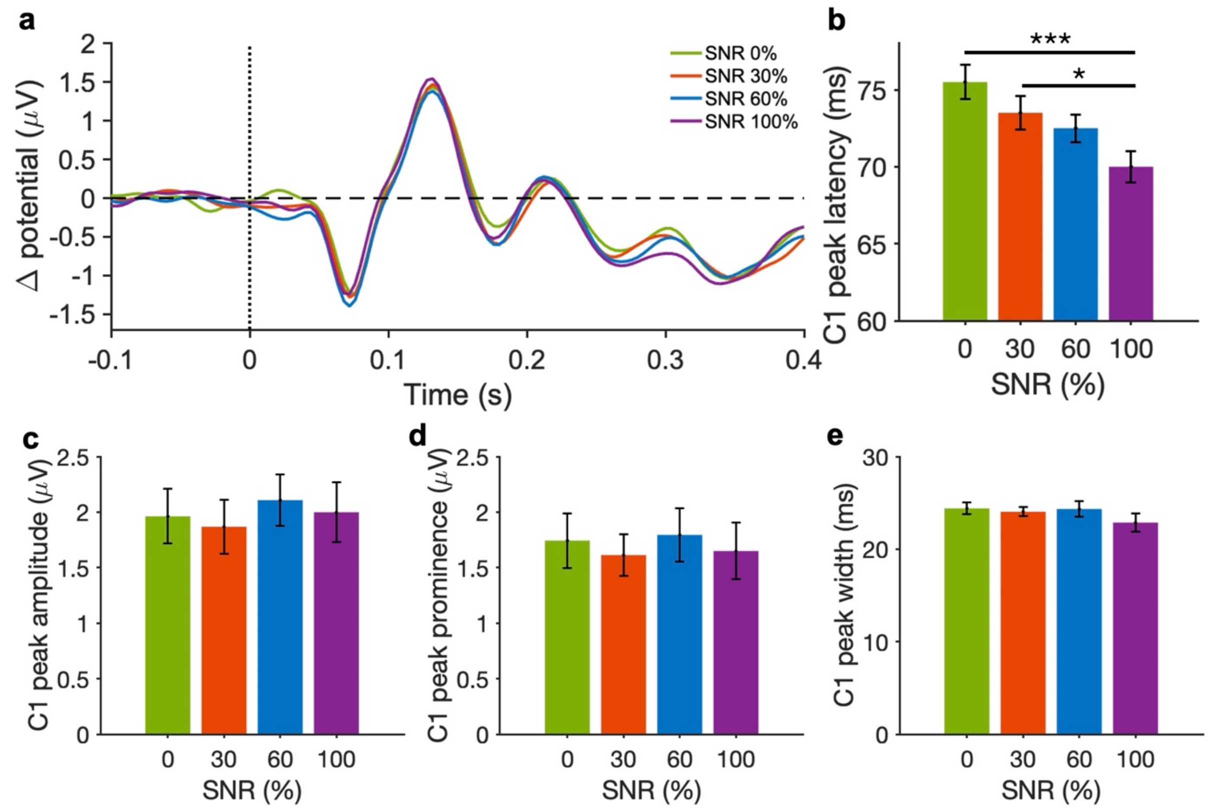

Supplementary Figure 5. C1 parameters analysis isolated for upper field stimulation. (a) Average VEP waves, including only the trials in which stimuli were presented in the upper visual field, across different SNR conditions. **(b)** C1 peak latency as a function of SNR levels. One-Way ANOVA showed a significant main effect of SNR on C1 latency (F (3, 60) = 7.32, $p < 0.001$, $\eta^2 = 0.39$ – large effect size). Post-hoc t-tests (Bonferroni correction) show a significant difference between latencies of stimuli with SNR 0% vs. 100% (t = 4.65, p < 0.001, d = 0.87 – large effect size, 95% CI [0.22, 1.53]), and SNR 30% vs. 100% (t = 2.79, p < 0.05, d = 0.5 – moderate effect size, 95% CI [-0.04, 1.1]). **(c)** C1 peak amplitude as a function of SNR levels (F (3, 60) = 0.5, $p = 0.52$). **(d)** C1 peak prominence as a function of SNR levels (F (3, 60) = 0.77, $p = 0.51$). **(e)** C1 peak width as a function of SNR levels (F (3, 60) = 0.42, $p = 0.73$). Asterisks mark statistically significant pairwise comparisons across SNR conditions: *p < 0.05, ***p < 0.001. Error bars are s.e.m. across participants.

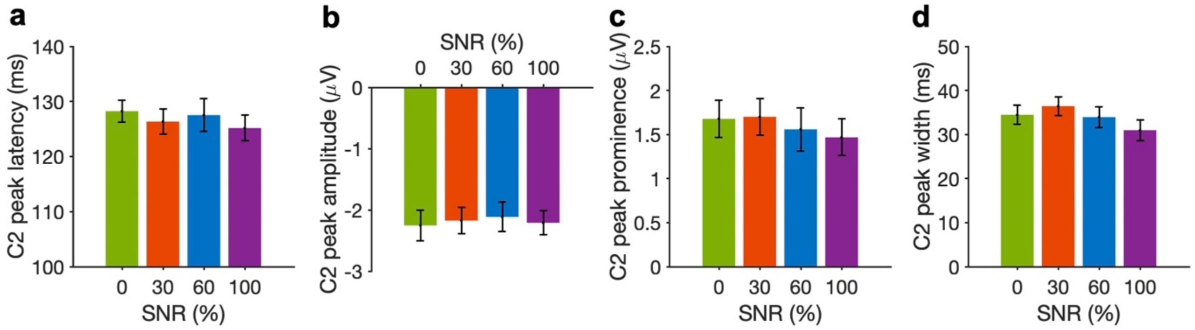

Supplementary Figure 6. C2 component parameters. (a) C2 peak latency as a function of SNR levels (F(1.8, 28.7) = 0.49; p = 0.6 – since sphericity assumptions were violated, the F statistics and associated degrees of freedom were corrected using the Greenhouse-Geisser method). **(b)** C2 peak amplitude as a function of SNR levels (F(3, 48) = 0.18; p = 0.9). **(c)** C2 peak prominence as a function of SNR levels (F(3, 48) = 0.86; p = .5). **(d)** C2 peak width as a function of SNR levels (F(3, 48) = 2.3; p = 0.09).

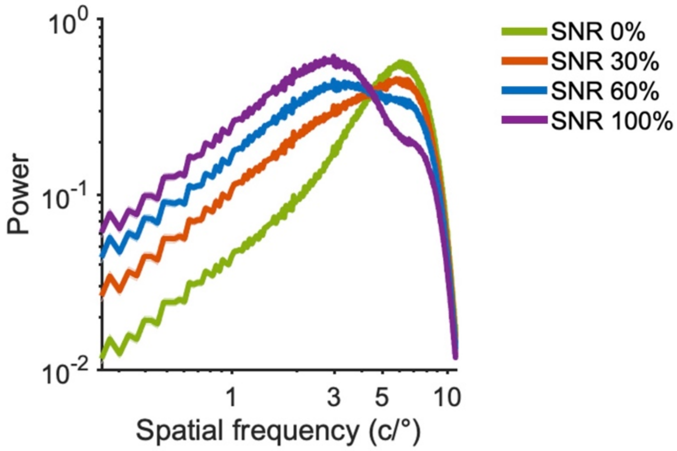

Supplementary Figure 7. Stimuli spatial frequency spectra. Average power spectral density estimated over the stimuli as in Figure 1C. The average spectrum of SNR 0% stimuli is statistically different from that of SNR 30% (D = 0.14, p < 0.001), SNR 60% (D = 0. 15, p < 0.001), and SNR 100% stimuli (D = 0.09, p < 0.1); the average spectrum of SNR 100% stimuli is statistically different from SNR 30% (D = 0.13, p < 0.001) and SNR 60% stimuli (D = 0. 13, p < 0.001); no significant difference emerged between SNR 30% and SNR 60% (D = 0.07, p > 0.5).
